# Treatment of localized prostate cancer in elderly patients: the role of partial cryoablation

**DOI:** 10.1007/s11255-023-03519-y

**Published:** 2023-02-21

**Authors:** Oscar Selvaggio, Marco Finati, Ugo Giovanni Falagario, Giovanni Silecchia, Marco Recchia, Andrea Alberto Checchia, Paola Milillo, Francesca Sanguedolce, Luca Cindolo, Gian Maria Busetto, Carlo Bettocchi, Luigi Cormio, Giuseppe Carrieri

**Affiliations:** 1 Department of Urology and Organ Transplantation, University of Foggia, Foggia, Italy; 2 Department of Urology, Bonomo Teaching Hospital, Andria (BAT), Andria, Italy; 3 Department of Diagnostic Imaging, University of Foggia, Foggia, Italy; 4 Department of Pathology, University of Foggia, Foggia, Italy; 5 Department of Urology, “Villa Stuart” Private Hospital, Rome, Italy; 6 Department of Urology and Renal Transplantation, University of Foggia, Viale Pinto 1, 71110 Foggia, Italy

**Keywords:** Cryoablation, Focal therapy, Prostate cancer, Minimally invasive surgery, Geriatric oncology

## Abstract

**Purpose:**

To evaluate oncological outcomes of partial gland cryoablation (PGC) for localized prostate cancer (PCa) in a cohort of elderly patients who required an active treatment.

**Methods:**

Data from 110 consecutive patients treated with PGC for localized PCa were collected. All patients underwent the same standardized follow-up with serum-PSA level and digital rectal examination. Prostate MRI and eventual re-biopsy were performed at twelve months after cryotherapy or in case of suspicion of recurrence. Biochemical recurrence was defined according to Phoenix criteria (PSA nadir + 2 ng/ml). Kaplan–Meier curves and Multivariable Cox Regression analyses were used to predict disease progression, biochemical recurrence- (BCS) and additional treatment-free survival (TFS).

**Results:**

Median age was 75 years (IQR 70–79). PGC was performed in 54 (49.1%) patients with low-risk PCa, 42 (38.1%) with intermediate risk and 14 (12.8%) high risk. At a median follow-up of 36 months, we recorded a BCS and TFS of 75 and 81%, respectively. At 5 years, BCS was 68.5% and CRS 71.5%.

High-risk prostate cancer was associated with lower TFS and BCS curves when compared with low-risk group (all *p* values < .03). A PSA reduction < 50% between preoperative level and nadir resulted as an independent failure predictor for all outcomes evaluated (all *p* values < .01). Age was not associated with worse outcomes.

**Conclusions:**

PGC could be a valid treatment for low- to intermediate PCa in elderly patients, when a curative approach is suitable in terms of life expectancy and quality of life.

## Introduction and aim of the study

Until a few years ago, treatment of localized prostate cancer (PCa) was primarily divided between active surveillance (AS) protocols versus radical treatments [1]. To date, focal therapy (FT) is emerging as a valid curative option, providing reasonable oncological outcomes, and limiting the typical side effects of radical surgery or external beam radiotherapy (ERBT) [2–4]. Based on these evidence, partial gland cryoablation (PGC) is gaining an increasing interest among PCa therapies due to its encouraging oncological and functional results [5, 6]. However, several main issues concerning FT are still under investigation. Specifically, due to medium-term outcomes and hardly comparable studies, no conclusive recommendation has been issued regarding specific indication of this treatment [7]. Indeed, European association of Urology (EAU) guidelines strongly recommend to only offer focal therapy within a clinical trial setting or well-designed prospective cohort study. Even if multiparametric magnetic resonance imaging (MRI) improved the accuracy of local staging of PCa [8], the usual multifocal aspect of the tumor remained one of the most critical point against these procedures [9].

When considering elderly population, EAU guidelines do not recommend any active treatment if the life expectancy is < 10 years, preferring a watchful waiting approach [1]. However, several studies have shown how deferred treatments could generate anxiety and distress in some patients, thus significantly affecting their quality of life (Qol) or determining discontinuation of the usual follow-up [10, 11]. Hence, PCa management in middle-old patients (75 years or older) is a hot topic among the uro-oncological community, since they potentially have a  > 10-year life expectancy but, due to comorbidities, they do not often result eligible for radical treatments [12] or could exhibit even worse side effects lead to continuous androgen deprivation therapy (ADT) [13].

Our retrospective study provided a monocentric experience in PGC for localized PCa, from a high-volume center with more than 10-year experience in cryotherapy. The aim of this study was to evaluate the oncological outcomes of elderly patients treated with hemi-gland or focal cryoablation for primary localized PCa. Then, according to our experience, we tried validating PGC as a therapeutic solution with reduced side effects for elderly patients who require an active treatment.

## Materials and methods

### Patients’ selection

**Fig. 1:**
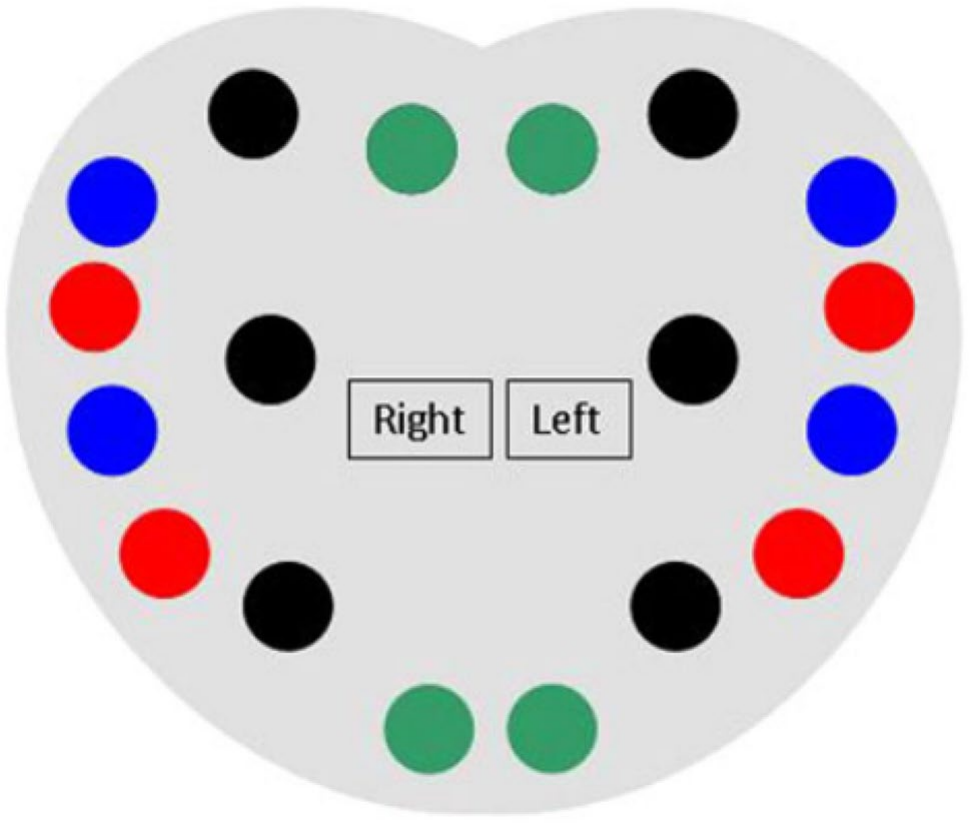
Location of the 18 cores in coronal projection. Location of the 18 cores in coronal projection. Black points represent the sextant cores, blue points the standard lateral peripheral cores, green points the paramedian peripheral cores and red points the additional lateral peripheral cores. Source: https://doi.org/10.1007/s00345-012-0989-8

We retrospectively collected data from 110 patients who underwent focal or hemiablative cryoablation from February 2013 to January 2021. All patients underwent preoperative examinations including digital rectal examination (DRE), serum-PSA test and transrectal ultrasonograpy (TRUS) to determine prostate volume. Therefore, they underwent a 18 cores standard transrectal biopsy following our Institutional biopsy scheme (Fig. 1) [14]. If pre-biopsy prostate MRI was available, MRI–US fusion biopsy was performed and two–three additional cores were taken from each suspicious MRI lesions.

Our Institutional cryoablation protocol included patients with EAU low- to intermediate-risk unilateral PCa who refused or were not eligible for radical treatments, low-risk PCa who refused AS or other radical treatments. Specifically, this cohort included patients with more than 70 years, good performance status and strongly motivated to undergo active treatment.

Indications for focal cryoablation were: maximum four contiguous unilateral positive cores or positive cores limited to target lesion found at MRI. The remaining cohort underwent hemiablative treatment, including highly selected patients with bilateral positive biopsy cores in whom there was low-volume disease in the controlateral lobe (defined as International Society of Urological Pathology—ISUP grade < 2 and maximum 2 cores involved).

Patients who underwent biopsy outside our Institution were excluded. After the procedure, patients were followed up at 30 days, then every 3 months for the first 2 years, every 6 months from the third to the 50th year and once a year until the 10th year. MRI imaging and eventual re-biopsy were strongly recommended at 12 months after PGC and in case of suspicion of disease recurrence.

### Surgical technique and outcomes’ measure

PGC was performed under spinal anesthesia by a single experienced surgeon (O.S) in a tertiary referral center with more than a hundred cryotherapy procedures per year for renal masses and PCa. Depending on lesion’s size and location, patients generally underwent two or three 10-min freeze–thaw cycles, using two–five 2.4 mm cryoprobes placed under transrectal ultrasound probe guidance. A preoperative saline solution was injected in the Denonvilliers’ fascia (Onik maneuver) to separate the prostate from the rectum. Focal freezing was obtained using an argon/helium gas-based system (Endocare, HeathTonics Inc., Austin, TX, USA); an urethral catheter was placed after the procedure (to be removed 7 days after the procedure) and the patient was discharged on post-operative day 1.

Presence of PC and its progression during follow-up were reported following Gleason Grade Group and ISUP grade [15, 16].

Biochemical recurrence (BCR) was assessed following Phoenix criteria (PSA nadir + 2 ng/ml) [17]. Treatment-free survival (TFS) was defined as lack of additional re-cryoablation, androgen deprivation therapy (ADT) or EBRT. Patients who underwent neoadjuvant or concomitant therapy were excluded from our cohort.

The Mann–Whitney and chi-square tests were used to compare continuous and statistical variables, respectively. Multivariable Cox Regression analysis and Kaplan–Meier curves were used to assess biochemical recurrence, treatment- and disease progression-free survival (BCS, TFS, PFS) adjusting for all available predictors. All analyses were performed using SPSS Statistics© 25 (SPSS, IBM Corporation, Armonk, NY, USA), considering *p* value < 0.05 at two-sided test as statistically significant.

## Results

Clinicopathological characteristics of patients are reported in Table 1. Median age was 75 years (IQR 70–79). 26 (23.6%) patients were treated with PGC, the remaining (76.4%) underwent hemiablative treatment. 20 (18.2%) patients showed bilateral positive cores at prostate biopsy and consequent underwent PGC only on the most clinically significant lesion. Baseline preoperative PSA and PSA density were 6.45 ng/ml (IQR 4.76–9.51) and 0.12 (0.09–0.19), respectively. Average prostate volume measured with transrectal ultrasonography or MRI was 50 cc (37–64). 54 (49.1%) patients eligible for PGC had a low-risk prostate cancer, while 42 (38.2%) and 14 (12.7%) showed an intermediate and high risk, respectively. 19 patients (17.2%) underwent MRI–US fusion biopsy and initial PIRADS score with respective ISUP grade is described in Table 1. With a median time to the PSA nadir of 3 months, the median PSA% reduction was 80 (56–92).

**Table 1:** Clinicopathological characteristics of patients treated with partial cryoablation

*N* = 110 Median follow-up, months (IQR): 36 (25–56)			
Age at surgery, (years), median (IQR)	76 (70–79)	Preoperative serum-PSA, ng/ml,median (IQR)	6.45 (4.76–9.51)
% positive biopsy core (median,IQR)	26 (11–38)	Cryotherapy technique, *n* (%)	
		Focal	26 (23.6)
		Hemiablative	84 (76.4)
Prostate Volume (cc), median (IQR)	50 (37–64)	PSA % reduction, median (IQR)	80 (56–92)
ISUP grade, *n* (%)		EAU risk, *n* (%)	
1	66 (60.0)	Low	54 (49.1)
2	22 (20.0)	Intermediate	42 (38.2)
3	12 (10.9)	High	14 (12.7)
4	9 (8.2)		
5	1 (0.9)		
PIRADS Score at pre-biopsy MRI, 19 (17.2%)		ISUP grade (MRI-fusion biopsy), *n *(%)	
		1	12 (10.9)
3	3 (2.7)	2	5 (2.6)
4	14 (12.7)	3	1 (0.9)
5	2 (1.8)	4	1 (0.9)
		5	0

### Oncological outcomes

At a median follow-up of 36 months (IQR 25–56), 27 (24.5%) patients having biochemical recurrences and 4 (3.6%) with suspicious lesions found at follow-up MRI (PIRADS 3b or more) underwent re-biopsy. 21 (19%) patients exhibit a clinical recurrence and subsequent additional treatment. In particular, 10 patients (9%) underwent retreatment with radical cryoablation and two (1.8%) radical prostatectomy. The remaining nine (8%) were treated with EBRT and/or ADT.

Disease progression was recorded in twelve patients (11%), seven of whom upgraded to a clinically significant (cs) PCa at follow-up biopsy as the remaining 4 were already ISUP ≥ 2 PCa.

**Fig. 2:**
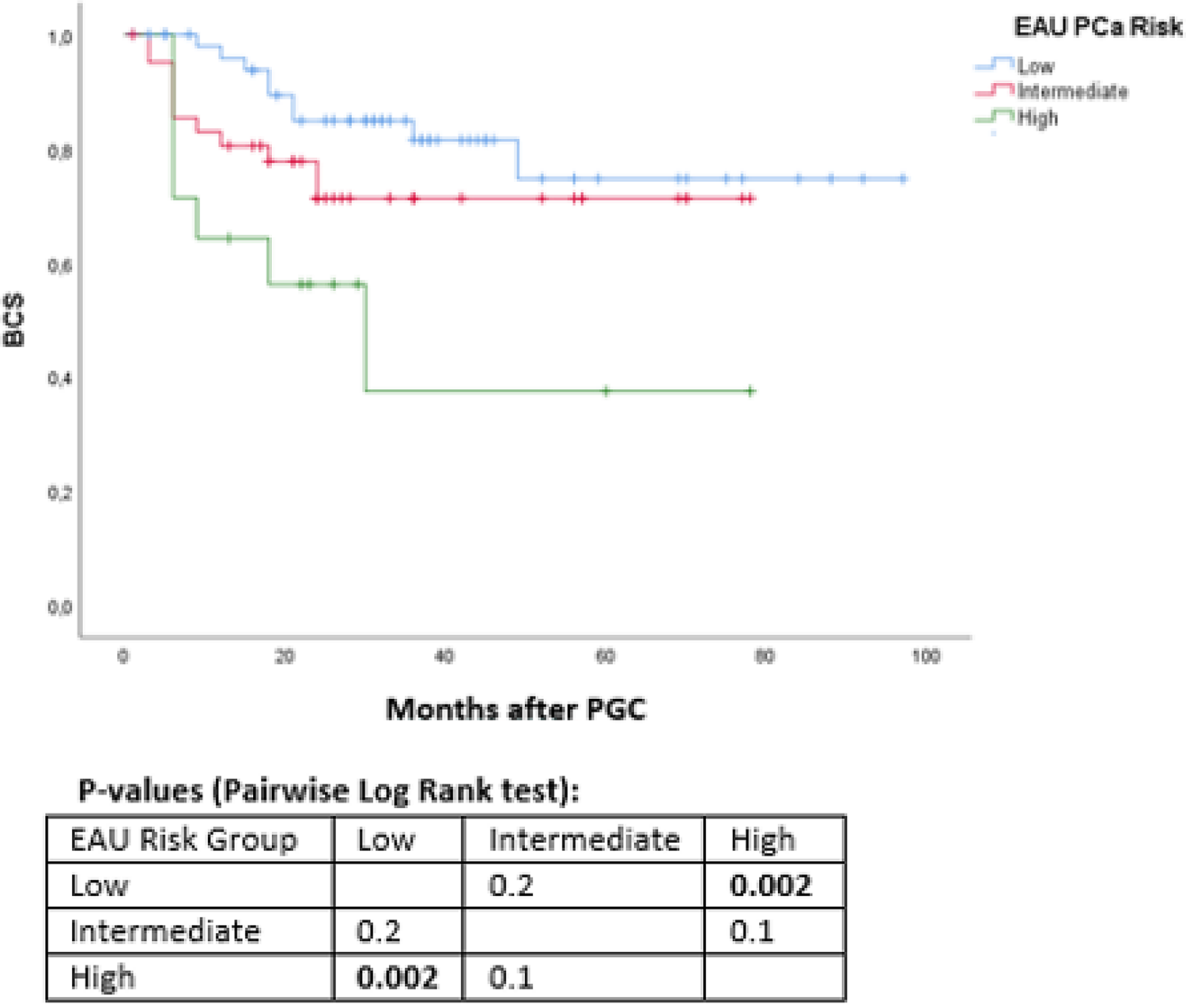
Kaplan–Meier curves addressing biochemical recurrence, stratified for EAU PCa risk groups [1]

**Fig. 3:**
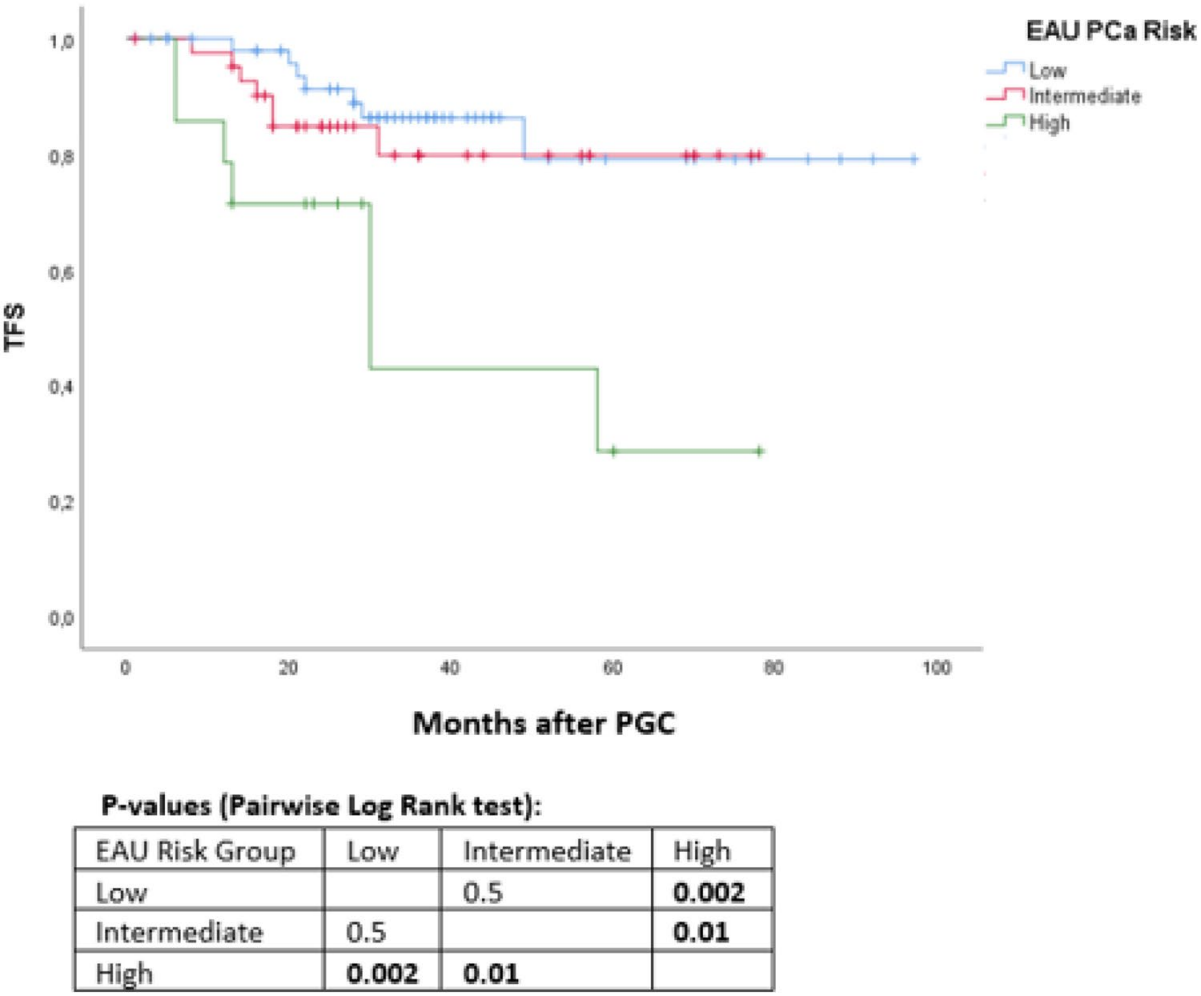
Kaplan–Meier curves addressing additional treatment-free survival, stratified for EAU PCa risk groups [1]

At Kaplan–Meier curves, high-risk PCa was associated with higher biochemical recurrence rate than low-risk (*p* value 0.002, Fig. 2). Moreover, patients with a high-risk PCa showed reduced TFS curves when compared to those with low and intermediate risk (all *p* values 0.01, Fig. 3).

Patients with a PSA% reduction < 50% after PGC showed reduced survival curves for all the three outcomes evaluated (all *p* values < 0.001). A prostate volume > 80 cc was associated with lower PSA reduction after treatment and consequent lower BRS (all *p* values < 0.04), albeit not being associated with higher clinical recurrence curve (Fig. 3).

**Fig. 4:**
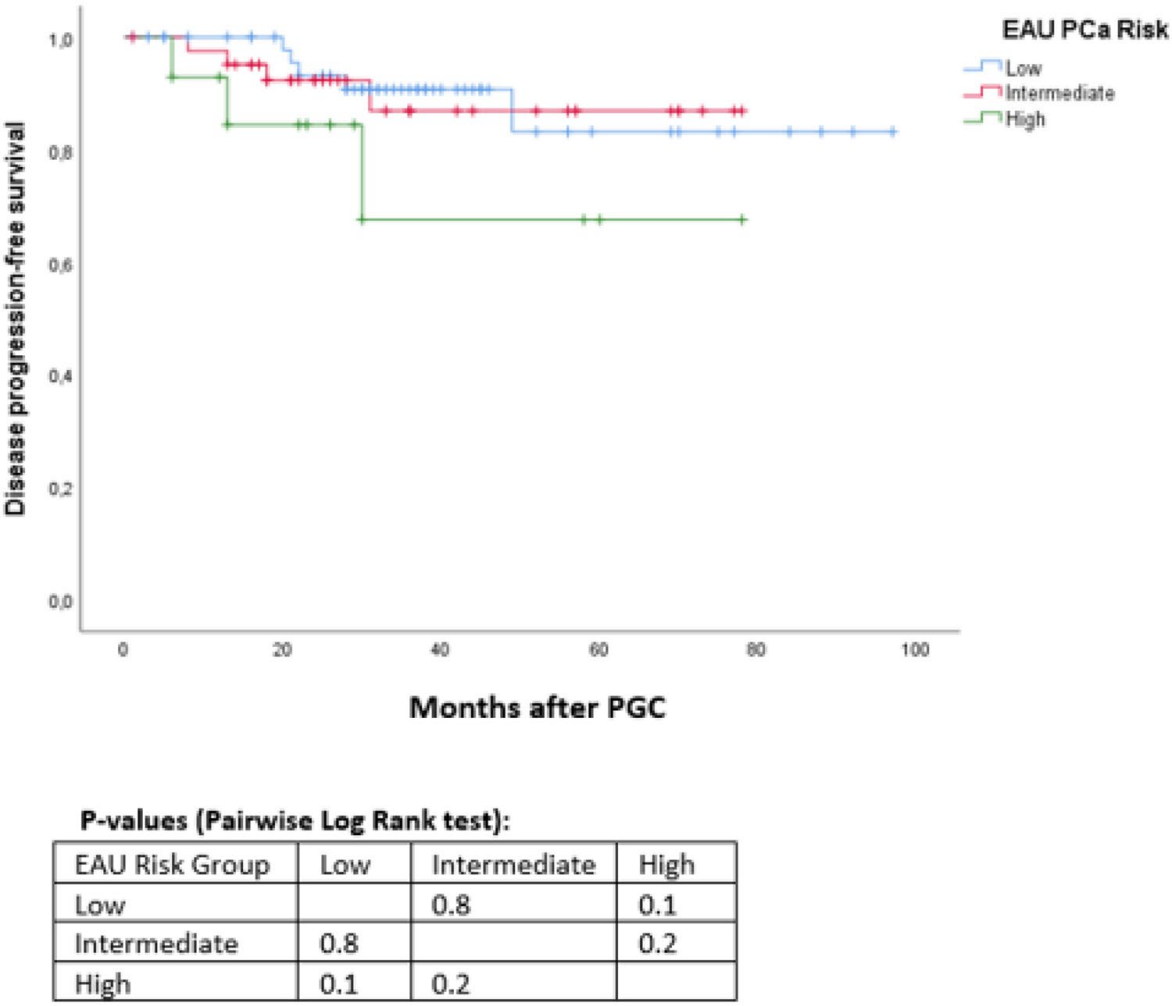
Kaplan–Meier curves addressing disease progression-free survival, stratified for EAU PCa risk groups [1]

No difference in terms of BCS and TFS curves were assessed comparing patients with unilateral versus bilateral lesion. Neither type of biopsy (standard versus MRI-fusion) nor initial PIRADS score impacted on the outcomes evaluated (Fig. 4).

At multivariable analyses (Table 2), a PSA reduction < 50% between preoperative value and PSA nadir resulted as an independent predictor for BCR, need of additional treatment and disease progression (all *p* values < 0.005). High-risk PCa was significantly associated with biochemical recurrence and additional treatment after PGC (all *p* values < 0.03) when compared with low risk. Age of patients and type of ablation (focal versus hemiablative) were not associated with worse outcomes. To date, none of the included patients developed metastatic PCa. At 5 years, BCS was 68.5% and CRS 71.5%.

**Table 2:** Multivariable Cox Regression analyses addressing BCR, TFS, disease progression after focal CA

Variables	BCR		TFS		Disease progression	
	HR (IC 95%)	*p* value	HR (IC 95%)	*p* value	HR (IC 95%)	*p* value
Age (years)	1.0 (0.95–1.13)	0.4	1.1 (0.98–1.20)	0.1	1.1 (0.97–1.29)	0.1
Preoperative PSA	0.9 (0.83–1.16)	0.8	0.9 (0.79–1.15)	0.6	0.8 (0.68–1.16)	0.4
PSA density	3.4 (0.61–19.89)	0.6	7.5 (0.08–74.35)	0.4	2.5 (0.02–33.01)	0.4
Prostate volume (cc)	1.0 (0.99–1.03)	0.4	1.0 (0.99–1.04)	0.4	1.0 (0.97–1.04)	0.8
TPC (%)	0.9 (0.95–1.03)	0.2	0.9 (0.90–1.01)	0.08	0.9 (0.88–1.02)	0.2
EAU risk						
Low	Ref		Ref		Ref	
Medium	2.8 (0.75–10.31)	0.1	3.1 (0.95–10.13)	0.06	2.3 (0.56–9.90)	0.2
High	3.2 (1.11–8.50)	0.03	5.2 (1.27–21.50)	0.02	4.3 (0.80–23.26)	0.08
PSA reduction						
> 50%	Ref		Ref		Ref	
< 50%	6.8 (2.71–16.99)	< 0.001	5.4 (1.95–14.67)	0.001	5.6 (1.57–20.21)	0.008

### Functional outcomes and surgical complications

According to Clavien–Dindo classification, no > 3a complications occurred during post-operative follow-up. One patient exhibited a scrotal edema, spontaneously resolved within 2 weeks. Two patients developed acute urinary retention (AUR) after catheter removal, requiring reinsertion of Foley catheter that was further removed within a month.

AUR occurred in other two patients after two months from PGC, thus they underwent a transurethral resection of the prostate (Clavien–Dindo 3a complication: 1.8%). Seven patients (6.3%) exhibited urge incontinence in the first 6 months after procedure and were treated with antimuscarinic drugs. Only two of them continued to experience the symptoms after 6 months from PGC. No follow-up data regarding sexual function were available.

## Discussion

In the present study, we report medium-term follow-up of patients treated with PGC at a single center.

Our study provides similar oncological outcomes results when compared with previous studies [18, 19]. Specifically, Oishi et al. found a BCS of 62%, with a treatment-free survival of 85% in a cohort of 160 patients with a median follow-up of 40 months [5]. The median age of our cohort was 10 years higher but we did not find any difference in oncological outcomes when we stratified the population according to age at treatment. Considering life expectancy increase and progressive population aging, the burden of PCa will require to reconsider treatment of localized disease in older men. Recent consensus statement from the International Society of Geriatric Oncology already recommended active treatment for fit elderly patients, according to their individual health status and not to chronological age [20].

Presence of other comorbidities and chronic conditions may affect natural history and biological aggressiveness of the tumor, resulting in limited treatment options and consequent poor prognosis.

As suggested by International Society of Geriatric Oncology (ISGO) since 2017, treatment of PCa in physically fit older patients should be the same as younger patients. However, higher risk of genito-urinary and rectal toxicity related either to surgery and EBRT should be taken into account in this population [20].

For these reasons, FT could emerge as a valid alternative for those patient with good general condition and adequate life expectancy [21].

No definitive indicators are available to establish the effectiveness of PGC [2–5, 22]. Biochemical recurrence is not infrequent after focal ablation, although patient’s prognosis and quality of life are mostly affected by disease progression and need of additional treatments. A > 50% reduction between preoperative PSA level and respective nadir seemed a valid predictor for PCa recurrence and progression in our cohort. In this perspective, we tried to evaluate which features could better predict PSA decrease after PGC. While a prostate volume > 80 cc was associated with a reduced PSA level reduction, patients with a high-risk PCa were more likely to exhibit a clinical recurrence when compared with those with an intermediate- or low-risk disease. Prostate volume must be carefully evaluated when performing FT, especially for HIFU and cryotherapy. Specifically, PGC could be hardly managed in small glands, due to higher risk of neurovascular bundle and urethra damages. On the other side, an optimal ablation of focal lesion may be more difficult in enlarged prostate, as shown in our study [19, 23].

Proper patient selection and the usual multifocal nature of PCa remains the two most controversial issues against FT and its applicability, since the key of this therapy is treating the so-called “Index lesion”, the largest and most aggressive lesion which should “drive” tumor progression and patient’s prognosis [24, 25]. In this perspective, multiparametric MRI and consequent fusion biopsy must be the standard diagnostic tool for FT, especially for targeted ablation [26]. Although our partial cryoablation experience started in 2013, long time before fusion biopsy resulted in a paradigm shift for PCa diagnosis, our standardized and reproducible biopsy scheme seemed to guarantee similar mid-term outcomes when compared with more recent FT series.

In the late years, we also enrolled patients with bilateral positive biopsy cores, thus only treating the side with more invasive and large-volume disease side and no statistical differences in BCS and TFS.

Although ours is a large series from a high-volume center with a proven experience in cryotherapy procedures, the sample size retrospectively collected is still relatively limited and do not overcome the current investigative nature of this treatment. Lack of standardized follow-up protocols have generated an extreme variability of outcomes reported, limiting possible comparison among the studies.

The vast majority of our patients exhibited low-risk disease and could not be considered as a proxy of a real population of older man with PCa, while FT impact on quality of life of our elderly patients was not assessed by any evaluation for QoL or HRQoL. Moreover, considering the median age of our cohort, not all patients who exhibited a biochemical recurrence underwent re-biopsy, since it would not have changed either prognosis or disease management. For these reasons, disease progression may be underestimated.

Conversely, the present paper has several strengths, reporting one of the biggest Italian monocentric series for PGC with a medium-term follow-up of 5 years after primary treatment. Surgery was performed by a single experienced surgeon with the same stabilized technique. All patients enrolled in the study were not previously treated for PCa and followed the same standardized check-up schedule after surgery.

## Conclusions

PGC could be a safe procedure in medium elderly patient, when a curative approach is suitable in terms of life expectancy and quality of life.

More studies with long-term outcomes are required to truly assess the efficacy of PGC and to establish definitive eligibility criteria for PCa focal treatment.

## Data Availability

No.
